# Case of Co-existence of Diptheria and Typhoid Fever

**Published:** 1883-09

**Authors:** 


CASE OF CO-EXISTENCE OF DIPHTHERIA AND TYPHOID FEVER.

Dr. C. E. Paget, F.R.S., Regius Professor of Physic in the University of Cambridge, describes the following case:

The recent illness of the Postmaster-General may add interest to the following case. The patient was Mrs. J. K., a married woman, about twenty-eight years of age, living in Manor street, Cambridge. Three days before her illness began one of her children had died of diphtheria, two of them having been affected. Mr. Carter, who attended them, had no doubt as to the diagnosis. The children had sore throat and exudation upon it.

When I first saw Mrs. K. (on December 14, 1861), she had been confined to her bed about a week. From Mr. Carter I learned that her illness had begun with sore throat, and that there had been small white diphtheritic patches upon the throat. When I examined it I could find none, nor any signs of diphtheria; but upon her abdomen were some of the rose spots characteristic of typhoid fever; and at the base of her right lung, to the extent of two or three inches, the percussion sound was dull, and small crepitation could be heard. She was feverish; her pulse was 130; her bowels loose. She was in the seventh month of pregnancy.

For six days she continued in much the same state, as an ordinary case of typhoid fever, with moderate pneumonic com-dlications; her bowels loose; her pulse above 120; her tongue dryish, and a general condition requiring wine and brandy. During these six days her throat remained free from diphtheritic appearances; but on the morning of December 20 it again became sore, and in the evening the uvula and soft palate were covered with a white exudation, the adjacent parts being bright red. Her pulse then became a little less frequent, falling to 116. Chlorate of potash was now prescribed in small, frequent doses, and next day tincture of perchloride of iron. On December 28, her urine contained albumen. The exudation, after its reappearance on December 20, was seen from day to day; it had a diphtheritic character, and was very extensive. It was still present, though somewhat reduced in extent, on January 2. When I saw her on January 5 it had been completely cleared off.

Early in January she began to suffer much from retching and vomiting. She was troubled also with cough. The right lung was consolidated at its base, but to a small extent only. The vomiting so persisted from day to day as to bring her into great peril. On January 20 the liquor amnii escaped. Active delirium now came on, and continued for upwards of twelve hours, when she suddenly aborted of a seven months' child, which lived half a day. The mother nearly died during the removal of the placenta, though scarcely any blood was lost. After labor was completed, the vomiting ceased, and she gradually recovered.

Mrs. K. had been nursed during her illness by her mother, Mrs. S., aged 58, who lived in the outskirts of Cambridge, in an isolated cottage within a large garden. On February 14, 1862, she took to her bed with typhoid fever. She had the ordinary symptoms: the rose spots, loose stools, etc. She went on favorably until March 13, when, after sitting up near an open door, she had rigors, ushering in double pneumonia and hæmorrhage from the bowels. She died on March 24.

The chief interest of Mrs. K.'s case is in the disappearance of the local signs of diphtheria, and their suspension for six days during the continuance of the typhoid fever, and then their reappearance and persistence for thirteen days. or more. This appears to me a fact, not perhaps contrary to what might be expected, but at least worth notice. Ic differs from what was reported in the case of Mr. Fawcett.—*British Medical Journal.*

